# The Abelian group structure of full factorial designs

**DOI:** 10.3389/fpsyg.2026.1764159

**Published:** 2026-05-20

**Authors:** Nicholas A. Altieri

**Affiliations:** Department of Mathematics, Bard Early College & Lincoln West Science and Health, Cleveland, OH, United States

**Keywords:** elementary p-groups, experimental design, full-factorial designs, logic gates, Systems Factorial Technology (SFT)

## Abstract

This study demonstrates formal connections between Abelian group theory and factorial experimental designs. I begin by showing that full-factorial designs are isomorphic to elementary p-groups. I continue with an analysis of the general group and subgroup structure of 3 × 3 full-factorial designs commonly utilized in detection studies, followed by extensions to more general but related designs in the subsequent section, namely, 3 × 3 × 3 designs. I show that the subgroup lattice of 3 × 3 designs decompose the experiment into single and double-target subgroups and a target-absent subgroup. An application of this endeavor is the identification of experimental strata, interactions, interaction contrasts, and fractional designs. By using examples from the reaction-time (RT) and non-parametric modeling literature, it is shown how comparisons across and within these subgroup structures can readily be used to derive predictions about human information processing. Relatedly, I provide predictions derived from subgroup analyses to increase statistical power and simplify data analyses. Finally, some suggestions are provided for analyzing more general designs.

## 1 Introduction

Group theory naturally describes structural symmetries inherent in randomized experimental designs. Generally, group theory helps identify patterns within designs, either by defining ***homomorphisms*** or by identifying simple relationships inherent in the group structure itself. In a seminal article, Bailey (1985; cf. 1981) provided many examples demonstrating how finite ***Abelian group*** theory facilitates the construction of experiments of varying degrees of complexity. Examples of methods for designing experiments with different factors and blocking structures, including Tjur, Latin-square, orthogonal, and poset block structures, and complete factorial designs.

One initial motivation for using group-theoretical tools was to provide a method ensuring that the different experimental factors remain independent of one another. Consequently, many design problems, particularly those related to avoiding confounds, have been simplified using such methods (cf. Bailey, 1985). For instance, experimental designs with multiple factors and levels were shown to decompose into direct sums, enabling the identification of patterns in certain subgroup decompositions. Doing so ensures that the conditions or plots are counterbalanced and randomized appropriately to avoid dependencies that may be overlooked. For example, designs with *n* factors and *k* levels within each factor typically aim for an equireplicate design in which each combination, or factor level, occurs an equal number of times. As experimental designs became increasingly complex since Fisher's (1935) study on randomized designs, (re)conceptualizing “randomization” within a group of permutations also proved beneficial. For instance, counterbalancing a design can be accomplished by considering the permutation group of *n*^*k*^ subplots, and randomly selecting elements from this group (Bailey, 1991).

While this research (e.g., Bailey, 1985) noted the Abelian group structure of factorial designs, this article here provides an in-depth description of why and how these structures arise in the presence of experimental factors with a prime number of levels. This endeavor is motivated by the paucity of discussion and utilization of group theory in experimental and quantitative psychology. Theorems and proofs are provided to demonstrate the importance of Abelian group theory in experimental design; this constitutes an important extension of previous research by specifically showing the isomorphism between factorial designs and p-elementary groups. I begin with an analysis of a simple full-factorial 3 × 3 detection designs in Section 2. Section 3 expands these foundational ideas to more complex 3 × 3 × 3 designs. Overall, the field will benefit from an informative tutorial on group-theoretical concepts related to common design structures.

Previous research has also overlooked how decomposing even simple experimental designs into subgroups can inform data analyses. Specifically, I show exactly how interaction contrast patterns and main effects arise within and across subgroups. This is accomplished in Section 4, which provides novel example applications that show how group elements act upon observable random variables, such as vectors of reaction times (RTs). Here, we also observe how subgroup analysis seamlessly fits into common experimental designs.

Much of this discussion is set within the framework of Systems Factorial Technology (SFT) (e.g., Little et al., 2017; Townsend and Nozawa, 1995). SFT is a theory-driven methodology that implements manipulations within factorial designs to identify information processing strategies: information gleaned from empirical reaction-time distributions is used to identify processing architecture (serial vs. parallel vs. coactive), capacity as a function of workload (comparing processing (dis)advantages when two targets vs. one target is present), and decision rule or logic gate. The examples describe how group-theoretic concepts intersect with the structure of SFT-type studies. Furthermore, future directions are suggested to simplify data analyses in SFT and other study designs via subgroup decomposition.

## 2 The group theoretic structure of 3 × 3 designs

As stated, the first purpose of this study involves providing a metatheory for the 3 × 3 full-factorial experimental design. I use the 3 × 3 nomenclature throughout to refer to cases in which two factors are presented at two levels, in addition to absent trials. Therefore, these designs could be said to contain three levels total, as absence is included. Importantly, this basic experimental design described in the following section remains especially relevant due to both its simplicity and utility: it has extensive applications in the reaction-time literature, which have historically used such information to make inferences regarding information processing (e.g., Little et al., 2017; Luce, 1986; Townsend, 1984; Townsend and Ashby, 1983).

**Example 2.1:** Consider a simple detection experiment requiring participants to respond Yes/No to the presence of stimuli. Stimuli may include flashes to the right or left of the center of a screen, two flashes presented in quick succession, tones played to the left and right ears or even a memorized list, and so on. For notational purposes, two factors A_*i*_ and B_*j*_, where *i* and *j* may correspond to the presence of a flash on the left and right sides of the screen, respectively. Second, let *i, j* ∈ {0, 1, 2} represent some physical level of the stimuli, such as duration or salience; let “2” indicate a high(er) level of salience (e.g., brighter flash) manipulated by the experimenter to yield a measurable change in the response variable. Naturally, “0” represents the absence of a stimulus. In this example, there technically exist three stimulus levels for each factor in “3 × 3” designs, with *n* × *n* stimulus levels. This design yields *n*^*k*^ possible conditions, where *n* represents the number of ordinal levels for each of the *k* factors. Assuming two experimental factors at three stimulus levels (absent = 0; along with 1, 2), we get 3^2^, *where n* = 3 *and k* = 2, yielding 9 experimental conditions enumerated by the set:

*S*_3 × 3_ = {*A*_0_*B*_0_, *A*_0_*B*_1_, *A*_0_*B*_2_, *A*_1_*B*_0_, *A*_2_*B*_0_, *A*_1_*B*_1_, *A*_1_*B*_2_, *A*_2_*B*_1_ , *A*_2_*B*_2_}. Critically, the experiment often presents several trials per condition in a fully randomized manner (cf. Townsend and Nozawa, 1995).

Two observations are in order before delving into group-theoretical structures from Example 2.1. First, when considering *S*_3 × 3_, one may notice that out of the nine elements, four distinct cycles can be formed if we consider the factor levels *i, j* ∈ {0, 1, 2} as an additive modulo 3; that is, a low level of salience is one ordinal level higher than the absent level in the experiment, and the high level of salience, “2,” is therefore now two levels higher than 0. Using this terminology, we may define a cycle common to group theory: 0 → 1 → 2 → 0. I utilize the common additive group notation, ⊕, to define the addition of experimental factor levels to say that high is the next “level up” from low in an ordinal fashion. (Here, the operator, ⊕, denotes the more abstract concept of “group” addition; examples include modular arithmetic, rotations of polygons in dihedral groups, and so on).

The reader may notice that each element, except addition on A_0_B_0_, forms cycles. To use just a couple of examples of addition occurring component-wise within each factor-level, observe that:


$$\begin{array}{l} A_{1}B_{2}⊕A_{1}B_{2}\;=\;A_{2}B_{4}\\ A_{2}B_{4}\;⊕A_{1}B_{2}\;=\;A_{3}B_{6\;}=\;A_{0}B_{0\;}\;\text{and}\;A_{1}B_{0}\;⊕\;A_{1}B_{0}\;=\;A_{2}B_{0}\\ A_{2}B_{0}\;⊕\;A_{1}B_{0}\;=\;A_{3}B_{0}=\;A_{0}B_{0}\;,\;\text{etc}. \end{array}$$

Critically, the group axioms hold under the operation of addition: (i) closure, (ii) existence of an additive inverse plus existence of the identity (0, 0) for each element, and (iii) associativity. Related to item (iii) mentioned above, observe that:


$$\begin{array}{l} A_{i}B_{j}⊕\left(A_{k}B_{l}⊕A_{m}B_{n}\right)\;=\;A_{i}B_{j}⊕\left(A_{k+m}B_{l+n}\right)\\ \;=\;A_{i+\left(k+m\right)}B_{j+\left(l+n\right)}\\ \;=\;A_{\left(i+k\right)+m}B_{\left(j+l\right)+n} \end{array}$$

via the associativity of addition. Therefore,


$$\begin{array}{l} \;A_{\left(i+k\right)+m}B_{\left(j+l\right)+n}\;=\;A_{i+k}B_{j+l}\;⊕A_{m}B_{n}\;\\ \;=\left(A_{i}B_{j}⊕A_{k}B_{l}\;\right)⊕\;A_{m}B_{n} \end{array}$$

This brings us another important observation: the group operator for addition is commutative since *A*_*i*_*B*_*j*_⊕*A*_*k*_*B*_*l*_ = *A*_*i*+*k*_*B*_*j*+*l*_ = *A*_*k*+*i*_*B*_*l*+*j*_ = *A*_*k*_*B*_*l*_⊕*A*_*i*_*B*_*j*_. After some simplification, we may write: (1, 2) ⊕ (1, 1) = (2, 3) = (2, 0) for *i, j* ∈ {0, 1, 2} with factor A specified by the left side and factor B by the right.

Combining the above observations yields the following lemma in group theory. Readers uninterested in formal mathematical details may skip the proofs, which are provided in Appendix A. Definitions for several mathematical terms, indicated by italicized bold letters throughout this study, are provided in Glossary.

**Lemma 2.1**: A full-factorial 3 × 3 design, specified by the set of elements *S*_3 × 3_, is isomorphic to an Abelian group of order 9 under the operation of addition.

This finding leads to the following known lemma about Abelian groups (e.g., Macdonald, 1968, Corollary 4.3 for a different proof):

**Lemma 2.2:** All groups of order nine are Abelian.

**Remark 2.1:** Related to discussion in Section 3, one might be tempted to assume that all groups of order 27 are Abelian. However, this is not the case. As for isomorphism classes of groups of order 27, four possibilities emerge, the first three of which are in fact Abelian: (i) the cyclic group ℤ_27_, (ii) the group comprised of direct products ℤ_9_ × ℤ_3_, (iii) and of course the p-elementary group discussed later ℤ_3_ × ℤ_3_ × ℤ_3_. Finally, (iv) the non-Abelian groups include the ***Heisenberg group*** of 3 × 3 upper triangular matrices over *F*_3_, and the ***extraspecial group***.

The following theorem gives a general result common to all 3 × 3 common full-factorial designs.

**Theorem 2.1**: The full-factorial identification design, whose elements are specified by the set *S*_3 × 3_, is isomorphic to the p-elementary Abelian group ℤ_3_ × ℤ_3_.

This paper shall refer to the experimental conditions using the notation derived from ℤ_3_ × ℤ_3_; for example, when factor level A is “low,” and B is “high,” I write (1,2) ∈ ℤ_3_ × ℤ_3_. I turn now to the subgroup analysis. The structure and interrelationships of the subgroups and their components will provide insight into the inherent relationships across experimental conditions.

### 2.1 Analysis of ℤ_3_ × ℤ_3_ subgroups

A subgroup **K** of group **G** is defined as a non-empty subset such that K contains the identity element, is closed under the group operation, and each element has an inverse (trivially, identity and **G** itself are subgroups): hence, a subset **K**⊆**G** that fulfills the group axioms.

Simple inspection reveals the non-trivial subgroups of ℤ_3_ × ℤ_3_ include the following four smaller groups, noting that they follow the group axioms under addition:

**M**_**1**_ = {(0, 0), (1, 0), (2, 0)}; **M**_**2**_ = {(0, 0), (0, 1), (0, 2)} and;

**D**_**1**_ = {(0, 0), (1, 2), (2, 1)}; **D**_**2**_ = {(0, 0), (1, 1), (2, 2)}

**Figure 1:**
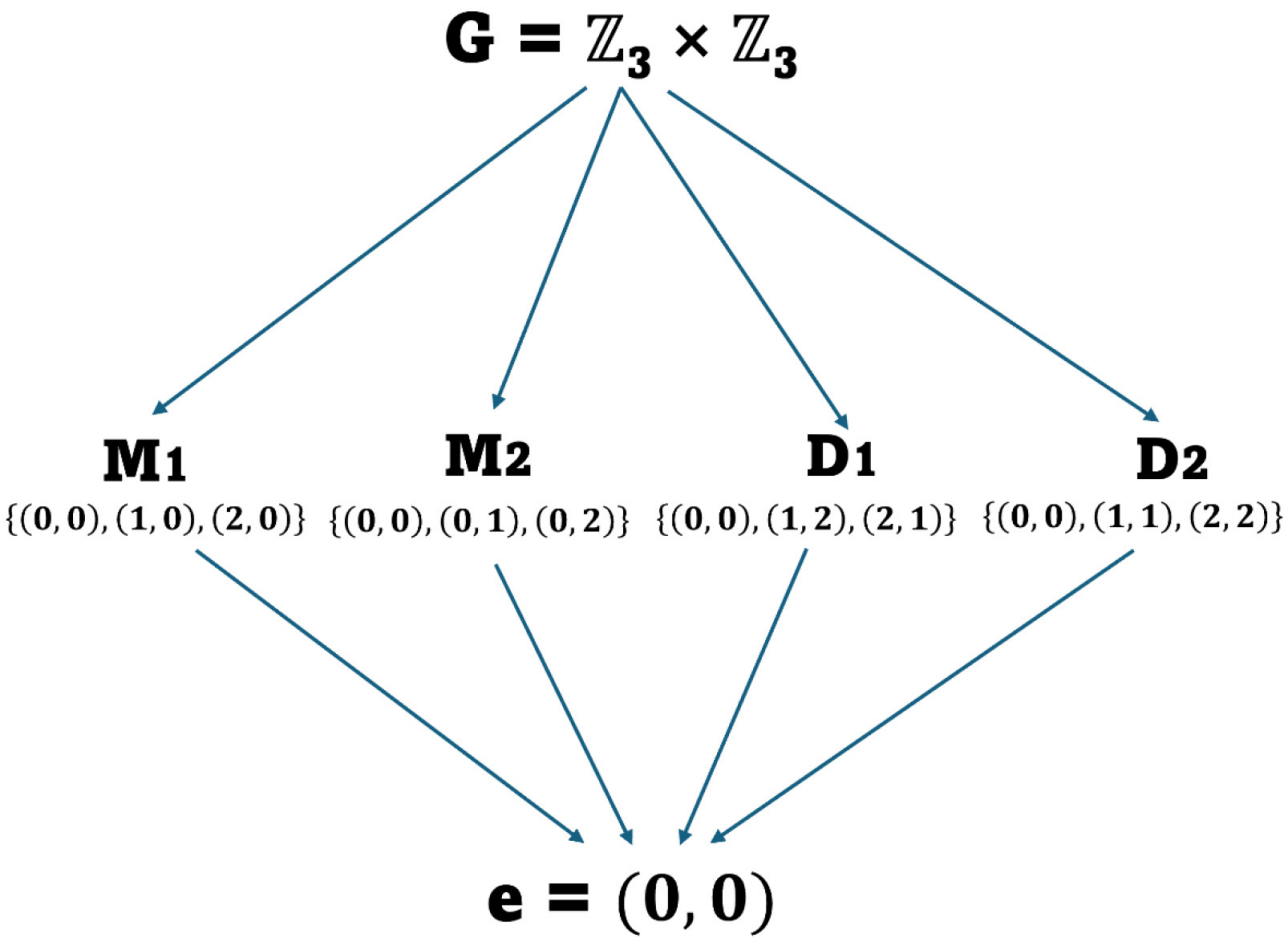
The subgroup lattice for ℤ_3_ × ℤ_3_. The arrows point to subgroups of a group.

The notation “**M**” (which refers to “marginal”) denotes the subgroups consisting of trial types, consisting of the presence of one “single-target” element; the subscripts “1” and “2” represent nominal indicators denoting different (“M”) subgroups (1 for factor A present, and 2 for factor B present). Likewise, “**D**” (again with subscripts) denotes the two subgroups consisting of double targets. The subgroup lattice is shown in Figure 1.

The reader may notice that the four proper subgroups do not contain any non-trivial subgroups. One interesting feature of ***G*** = ℤ_3_ × ℤ_3_ is the intersection of its maximal subgroups, which forms the ***Frattini group*** Φ(**G**), contains only the identity element **e** (see Bechtell, 1965, for discussion of elementary groups). This may also be observed directly by noting that, by Lagrange's theorem, the order of a subgroup must divide the order of the group, where *p* = 3 divides 9. Additionally, each subgroup contains its own generator of order 3. Therefore, each maximal subgroup and their intersections must be disjoint except for the identity element. Interestingly, when observing the subgroup lattice structure, we note that for any subgroup **H** of an elementary group, there exists another subgroup **K** such that **HK** = **G** (e.g., **M**_**1**_ ⊕ **D**_**2**_**=** **G**). Thus, when combined via direct sum, they generate the entire group. This observation is relevant here for this design, having implications for data analyses since plots containing two targets fall into subgroups separate from those containing one single target.

## 3 Beyond 3** × **3 designs

A group theoretical account of full-factorial designs must extend beyond the simple, albeit highly useful, 3 × 3 cases. A myriad of other experimental designs have proven useful. These include simple present-absent judgments of one or more factors, such as items in a memory set, including those ≅ℤ_3_ × ℤ_3_ assuming two factors without saliency manipulation, as well as designs containing three factors at three levels—namely, two levels of saliency plus absent trials (≅ℤ_3_ × ℤ_3_ × ℤ_3_; e.g., Bailey, 1985; Winer et al., 1971).

### 3.1 Subgroup structure of 3 × 3 × 3 full-factorial designs

I now discuss the group-theoretical structure of 3 × 3 × 3 full-factorial designs **G** ≅ℤ_3_ × ℤ_3_ × ℤ_3_ with three factors at three levels yielding 27 total conditions. The structure specified by 3 × 3 × 3 full-factorial design is more complex than the two-factor 3 × 3 design.

**Example 3.1a**: One may easily generalize 3 × 3 × 3 cases by considering three factors each presented at two levels of saliency, plus target-absent cases. These could, again, involve simple detection requiring Yes/No responses. This may include a detection design presenting a flash using two saliency levels in up to three positions on a computer screen, or a modified detection design requiring the identification of a flash on the computer screen, and the detection of a signal presented to the left or right ear, etc.

**Example 3.1b**: Consider a Sternbergian (e.g., Sternberg, 1969a,b) style memory task involving the presentation of a stimulus set followed by a probe and a Yes/No response. Stimuli can include the presentation of 0 (catch trials), one, two, or three letters at two salience levels. Salience manipulations might include factors such as stimulus clarity or length of presentation.

First, as shown in **Theorem 3.2**, 3 × 3 × 3 full-factorial designs with two saliency levels plus absence are isomorphic to ℤ_3_ × ℤ_3_ × ℤ_3_–the Abelian p-elementary group with 27 elements of order 3. Unlike ℤ_3_ × ℤ_3_ with only 9 elements, subgroup identification becomes more complex. Here, we are aided by the general simplicity of Abelian elementary groups and Lagrange's theorem: For a group of 27 elements, each non-trivial subgroup must be of order 3 or 9. We are further aided by Sylow's theorem dictating that the number of subgroups of order 3^n^ must be ≡1 *mod*(*p*). Also, since ℤ_3_ × ℤ_3_ × ℤ_3_ constitutes a 3-dimensional vector space, the subgroups of order 3 = 3^1^ are its one-dimensional linear subspaces: the number can be enumerated using the ***Gaussian binomial*** ***coefficient*** ${\left(\begin{array}{c} n\\ k \end{array}\right)}_{q}$. Let *n* be the dimension of the space (number of factors = 3), *k* be the dimension of the subspace (dim = 1) and *q* the number of factor levels (*q* = 3). This yields $\frac{3^{3}-1}{3^{1}-1}=\frac{26}{2}=13$ total subgroups of order 3. Identical logic using the Gaussian binomial, with subspace dimension 2 (dim = 2), shows that there are also 13 subgroups of order 9.

Subgroups of order 3 constitute a 1-dimensional subspace of the 3-dimensional vector space. This subspace is the kernel of the (non-zero) linear functional *f*(*x, y, z*) = *ax*+*by*+*cz* = 0. Computing (a, b, c) mod(3) over a representative set of the 26/2 = 13 non-zero vectors (modulo the scalar multiple of 2) yield each subgroup of order 3. Likewise, subgroups of order 3^2^ = 9 constitute a 2-dimensional subspace of the 3-dimensional vector space and represent the kernel of the linear functional. The order-3 and order-9 subgroups are displayed in Appendix B. The subgroups of order 3 can also be easily computed by considering all pairs of non-zero vectors adding to 0 modulo 3. These are organized according to 1, 2, and 3-***heavy subgroups*** (containing the presence of 1, 2, or 3 factors, respectively). These should prove useful for identifying fractional designs that test main effects or interactions via designs of different resolutions (see Bailey, 1985).

Next, for subgroups of order 9, the first 3 listed are those isomorphic to ℤ_3_ × ℤ_3_. These designs should be of particular interest, especially when considering 1/3 fractional replicate designs in detection or identification experiments; that is, when it is of interest to assay the main effects and interactions from two of the three factors. The other subgroups contain useful information relevant for 1/3 fractional designs for testing other main effects, interactions, and higher order interactions. Finally, as was the case with the 3 × 3 design, we again observe that the Frattini group, formed via the intersection of the maximal subgroups of order 9, contains only the identity element (0, 0, 0).

### 3.2 Other factorial designs

Some general observations will help facilitate future discussion on these topics. First, as stated in Section 2, full-factorial designs with *k* factors and *n* levels, containing *n*^*k*^ total trial types are isomorphic to a p-elementary group, when *n* equals the same prime number p for each factor (i.e., *p*^*k*^). This means that the set of experimental conditions S specified by a p × p × … × p design with *k* factors ≅ℤ_*p*_ × ℤ_*p*_ × ... × ℤ_*p*_ (*k times*).

Of course, each factor might not necessarily contain the same number of levels. 2 × 1 and 3 × 2 designs, for instance, appear useful for simple detection or for full-factorial identification studies. Instantiations of the former might involve a detection task presenting a letter at two levels of saliency via brightness or clarity manipulations, and a tone at one level. These trial-types may be specified by the direct product isomorphically described by ℤ_3_ × ℤ_2_ = {(0, 0), (1, 0), (2, 0), (0, 1), (1, 1), (2, 1)}. Importantly, cases in which the levels *m* and *n* are relatively prime in ℤ_*m*_ × ℤ_*n*_ do not yield an isomorphism to p-elementary groups. This is because when gcd(m, n) = 1, ℤ_*m*_ × ℤ_*n*_≅ *Z*_*mn*_, meaning the isomorphism is now with the cyclic group of order *mn*. One may notice that the entire group ℤ_3_ × ℤ_2_≅ ℤ_6_ is generated by the repeated addition of the element < (1,1)>. Consequently, the subgroup lattice structure differs from that of elementary groups, with one feature of these designs being that they can be decomposed into their separate cyclic subgroups of orders *m* and *n*, which may be useful for within-factor comparisons.

For general *m* × *n* × … designs in which *m* and *n* are neither identical primes, nor relatively prime to one another, an entirely different situation arises. Consider the following example: an experimental design with two factors, the first at 4 levels and the second at 4 levels, isomorphic structurally to ℤ_4_ × ℤ_4_ with 4^2^ = 16 total trial types. While this is not a p-elementary group but rather p^2^, it does contain within it a p-elementary subgroup, namely ℤ_2_ × ℤ_2_, which is also useful for examination.

Putting everything together leads to the following proposition, which represents a generalization of **Theorem 2.1**.

**Theorem 3.2**: Consider the full-factorial design A_i_ × B_j_ × … × C_k_ ≅ to ℤ_*m*_ × ℤ_*n*_ × ... × ℤ_*r*_. By Lemma 2.1, it can be shown to be isomorphic to an Abelian group, namely, a direct product *Z*_*m*_ × *Z*_*n*_ × ... × *Z*_*r*_. Assume factor levels *m, n*, and *r* are integers not relatively prime. Then, there exists a p-elementary subgroup ℤ_*p*_ × ℤ_*p*_ × ... × ℤ_*p*_ , with an identical number of factors as ℤ_*m*_ × ℤ_*n*_ × ... × ℤ_*r*_.

**Example 3.3**: One functional application of experimental designs with *k*-factors ≅ℤ_3_ × ℤ_3_ × ... × ℤ_3_ (*k times*) involves computing interaction contrasts (in detection studies) when all “targets” are present. This endeavor is useful for systems identification using non-parametric modeling approaches, such as determining whether targets are processed in parallel or serial, and whether the decision rule is first-terminating (OR logic gate), or exhaustive (AND logic gate) (see Townsend and Nozawa, 1995). Yang et al. (2013) demonstrated the existence of survivor interaction contrasts (i.e., distributions of RTs) by recursively embedding double differences within double differences across trial types when all targets were present.

I noted previously that the elementary group ℤ_3_ × ℤ_3_ yields four double-target trial types spread across two subgroups, **D**_**1**_ and **D**_**2**_. This arises from the fact that in p-elementary groups, all elements have the same order which is a prime number p. This leads to the following proposition.

**Proposition 3.4**: The number of trial types in which all targets are present (in p-elementary groups) is (p – 1)^k^.

**Remark 3.5**: One caveat relates to the empirical practicality of carrying out generalized full-factorial designs. To obtain enough trial types to compute empirical cumulative distribution or survivor functions, *n* and *k* must be “small enough.” For example, ℤ_3_ × ℤ_3_ × ℤ_3_ specifies *n*^*k*^ = 3^3^ which equals 27 trial types, while ℤ_4_ × ℤ_4_ × ℤ_4_ specifies *n*^*k*^ = 4^3^ or 64 trial types. The latter case becomes difficult for practical purposes if we assume the existence of multiple trials per condition.

To summarize, when considering the set of conditions from generalized full-factorial designs under the assumption that factor-levels represent an ordinal salience manipulation, three possible scenarios emerge: (i) The experimental design is isomorphic to a cyclic group ℤ_*n*_ when the number of levels across factors are relatively prime, (ii) the design has a subgroup isomorphic to a p-elementary group, or (iii) the design itself is isomorphic to a p-elementary group.

## 4 Applications and general discussion

This section presents applications showing how comparisons of experimental conditions and interaction contrasts, identifiable by subgroups, can help identify designs and provide avenues for novel data analyses. However, describing how group elements as experimental conditions act upon random variables will prove beneficial prior to addressing these applications. Experimental conditions, manipulated by the experimenter by virtue of the design, yield a random vector of outputs: outputs become manifest as observables in the form of a random vector of RTs or binary responses such as accuracy, etc.

### 4.1 . Groups acting on random variables: reaction-time distributions

Let **T** denote reaction time (RT) as the observable random variable. When considering processing times for individual components, assign random vector **T**_A_ for processing time on component A and **T**_B_ for processing time on component B. Decomposition rules relevant to many designs include those associated with OR and AND logic gates, or strictly serial processes in which inputs are processed sequentially (Dzhafarov and Schweickert, 1995): *T*: = *min*(*T*_*A*_, *T*_*B*_) for parallel first terminating (OR gate), *T*: = max(*T*_*A*_, *T*_*B*_) for parallel exhaustive (AND gate) processing; for the serial cases in which case inputs are processed sequentially: *T*: = *T*_*A, B*_ and *T*: = *T*_*A*_+ *T*_*B*_ for first-terminating and exhaustive processing, respectively (with the notation := denoting “distributed as”).

**Example 4.1:** For simplicity, consider the elementary group ℤ_3_ × ℤ_3_ placed into one-to-one correspondence with the conditions of a prototypical 3 × 3 full-factorial detection design. Per Example 2.1, consider a detection experiment with either an OR or AND decision rule with *n* experimental trials per condition. The ***probability space*** with for such an experiment, producing all possible random vectors of response-times across the experimental conditions with a probability measure **P** that is absolutely continuous with respect to the Lebesgue measure λ on *R*^+^, can be described via the following decomposition notation: $\left(\Omega ,\;\text{F},\text{P}\;\right)\;=\;{⊕}_{\left(i,j\right)\in Z_{3}}\left(R^{n+},\;B{\left(R^{+}\right)}^{⊗n},\;P^{⊗n}\right)$. Intuitively, this direct sum notation and product notation specify the possible 9-dimensional vector space and its subspaces consisting of RTs (positive real numbers) that would be obtainable in the described experimental set-up. Table 1 shows the response mappings for key experimental designs and decision rules.

**Table 1:** Common decision rules using the 3 × 3 experimental design.

Targets A	Target B	Group element	OR	AND	XOR	Identification
Present	Present	(*i, j*), *i, j*≠0	Yes	Yes	No	“1”
Present	Ø	(*i*, 0), *i*≠0)	Yes	No	Yes	“2”
Ø	Present	(0, *j*), *j*≠0	Yes	No	Yes	“3”
Ø	Ø	(0, 0)	No	No	No	“4”

These include the OR, AND, XOR, and identification designs, with “correct” responses listed in the corresponding columns. The other columns represent whether each target is present or absent, and the corresponding group element.

I now describe how groups may act upon sets to produce the random output vector **T** of RTs for some arbitrary experimental condition using Example 4.1. For continuous functions, consider the mapping *f*:*G*→*V* defined as *g*↦*f*(*s, g*) and letting *T* = *f*(*s, g*). **G** represents a group, while **V** represents a random vector whose components are elements of *R*^+^. Let *f* represent the probability density function, *s* a specific source of variance within an individual participant, and *g* the experimental condition ∈ **G**. We may define group element *g* = ((*i, j*):*i, j* ∈ *Z*_3_) and (*s*, (*i, j*)). Other specifications may also include common sources of variance, denoted by C, *f*(*C, s, g* ).

Integrating the density function yields the probability distribution function(s):


$$\begin{array}{l} OR\;Rule:\;P\left[T_{i}\leq t\cup \;T_{j}\leq t,\;|\;\left(i,j\right),\;s\;=\;s_{k}\right]\\ AND\;Rule:\;P\left[T_{i}\leq t\cap \;T_{j}\leq t\;|\;\left(i,j\right),\;s\;=\;s_{k}\right] \end{array}$$

These indicate the probability that processing on components A and B has terminated by time *t* when stimulus configuration (*i, j*) is presented under OR and AND rules (assuming a subject-specific value for specific source of variance, *s*_*k*_). Also, while the empirical joint distribution for **T**_i, j_ is directly observable, the marginal distribution for each component is not directly observable (consider the OR decision rule: any given observed RT does not indicate whether processing has terminated on component A, B, or both by time *t*). Simplified probability distribution functions without assuming a specific source of variance (cf. Example 4.2) can be written as:

*P*[*T*_*A*_ ≤ *t* | *A*]; this represents the probability that processing terminates by time *t* given the presence of factor A alone (a stimulus at any level in location A or B, respectively), and *P*[*T*_*A*_ ≤ *t* ∪ *T*_*B*_ ≤ *t* | *AB*] indicating the probability that processing terminates by time *t* on component A OR component B, given the presence of stimuli in both locations (A and B at any level). Finally, 1 – *P*[“”] denotes the survivor function.

The following examples reveal how subgroup delineations shown in Figure 1 are informative for data analyses and drawing conclusions regarding model-theoretic analyses:

**Example 4.2:** One area of research in the SFT literature involves comparing RT distributions in double target (D) conditions to parallel-independent model predictions derived mathematically from marginal (M) single-target conditions (e.g., Little et al., 2017; Howard et al., 2021; Townsend and Altieri, 2012; Little et al., 2015). A measure, commonly known as workload or processing “capacity,” assesses the relative efficiency of the system by comparing RT distributions involving two targets vs. the presentation of one target at a time. For example, the capacity derivation for the OR rule given originally by (Townsend and Nozawa 1995) is expressed as:


$$\begin{array}{r} P\left[T_{A}\leq t\;\cup \;T_{B}\leq t\;|\;AB\right]=P\left[T_{A}\leq t\;|\;A\right]\;+P\left[T_{B}\leq t\;|\;B\right]\\ \;-P\left[T_{A}\leq t\;|\;A\right]\cdot \;P\left[T_{B}\leq t\;|\;B\;\right] \end{array}$$

Using survivor functions:


$$\begin{array}{l} 1-P\left[T_{A}\leq t\;\cup \;T_{B}\leq t\;|\;AB\right]=1-(P[T_{A}\leq t\;|\;A]\;\\ \;+P[T_{B}\leq t\;|\;B\;]\;-\\ P[T_{A}\leq t\;|\;A]P[T_{B}\leq t\;|\;B])=(1-P[T_{A}\leq t\;|\;A])(1\\ \;-P[T_{B}\leq t\;|\;B\;]) \end{array}$$

Now, taking the negative logarithm transformations for the purpose of obtaining integrated hazard functions yields:


$$\begin{array}{l} -log\left(1-P\left[T_{A}\leq t\;\cup \;T_{B}\leq t\;|\;AB\right]\right)\;=\\ -log\left(\left(1-P\left[T_{A}\leq t\;|\;A\right]\;\right)\left(1-P\left[T_{B}\leq t\;|\;B\;\right]\right)\right)\\ =\;-log\left(1-P\left[T_{A}\leq t\;|\;A\;\right]\right)\;-\;log\left(1-P\left[T_{B}\leq t\;|\;B\right]\right) \end{array}$$

Since the negative signs cancel, we arrive at the following capacity coefficient *C*(*t*):


$$C\left(t\right)=\frac{log(1-P\left[T_{A}\leq t\cup T_{B}\leq t|AB\right]}{\log\left(1-P\left[T_{A}\leq t|A\right]\right)+log\left(1-P\left[T_{B}\leq t|B\right]\right)},$$

where *C*(*t*) = 1 under parallel independent-race model predictions with an OR logic gate.

More recently, Howard et al. (2021) extended these ideas to include full-factorial identification designs (Table 1); their modified capacity coefficient compares RT distributions obtained from the double-target and target-absent conditions to the distributions obtained from single-target conditions. In each case, these delineations are revealed by the subgroup structure shown in Figure 1.

**Example 4.3**. Related SFT research has long utilized interaction contrasts (cumulative distribution or survivor) among the double-target conditions or “subgroups.” In doing so, empirical survivor (or distribution) functions may be obtained from each of the double-target conditions specified by D_1_ and D_2_, and the interaction contrast is subsequently carried out. Critically, the shape of the interaction contrast function provides information regarding the architecture and the logic gate (cf. Townsend and Nozawa, 1995).

Schweickert et al. (2000) also proved the following four identities regarding interaction contrasts, denoted by *c*(*t*) for parallel vs. serial architecture and OR/AND logic gates. Define each double-target probability distribution function as *G*_*ij*_(*t*) = *P*[**T**_**ij**_ ≤ *t* (*i, j*)] *s*.*t*. *i, j* = 1 *or* 2 correspond to the salience level of components A and B in a 3 × 3 full-factorial detection design. For *c*(*t*) = [*G*_11_(*t*)−*G*_12_(*t*)]−[*G*_21_(*t*)−*G*_22_(*t*)], we get for all *t* ≥ 0 assuming stochastic independence and dominance (cf. Schweickert et al., 2000; also Townsend and Nozawa, 1995):

1. Parallel (concurrent) OR: *c*(*t*) ≤ 0
2. Parallel (concurrent) AND: *c*(*t*) ≥ 0
3. Serial (sequential) OR: *c*(*t*) = 0
4. Serial (sequential) AND: $\int_{0}^{t}c\left(t\right)dt\geq 0,\;\int_{t}^{\infty }c\left(t\right)dt\leq 0,\;where\;\int_{0}^{\infty }c\left(t\right)dt\;=\;0$.

[These predictions correspond to proofs by Townsend and Nozawa (1995), with the difference being that the latter's predictions are reversed in polarity due to the use of survivor functions rather than distribution functions].

**Remark 4.3:** Dzhafarov et al. (2004) derived predictions identical to 1–4 for cases with distribution functions conditioned on a specific value C = c, with C representing the common source of variance (note the authors used “R” instead of “C”). Here, cumulative distribution functions can thus be re-expressed as *G*_*ij*_(*t* | *c*) = *P*[**T**_**ij**_ ≤ *t* (*i, j*), *C* = *c*], with the interaction contrast now being *c*(*t* | *C* = *c*). This allows for stochastic interdependence across random vectors of RTs while assuming that RTs in each condition are only influenced by the factors present in that condition.

### 4.2 Future directions

These examples show that subgroup structure nicely describes the breakdown of experimental conditions. One avenue using Example 4.3 involves developing more powerful architecture and decision rule analyses by identifying key dominance relationships, both among and within subgroups. One important suggestion for future research is to derive parallel and serial models (OR and AND rules) under the assumption of stochastic dominance (i.e., high salience “2” leads to faster identification than low salience “1”.

**Example 4.4:** The purpose of this example is to show that data analysis can be substantially simplified by accounting for subgroup structure.

(i) Importantly, for OR decision rules, parallel models should predict that **D**_**2**_ predictably leads to faster RTs compared to both the **M**_**1**_ and **M**_**2**_ single-target conditions. These predictions can be derived from stochastic dominance assumptions and parallel-race model predictions (e.g., Miller, 1982).
(ii) Parallel AND predictions appear more difficult to derive since “No” responses are required for target absent, **e**, and single target **M**_**1**_**/M**_**2**_ trials, and “yes” only for the double target. We are, however, aided by recent research reported by Howard et al. (2021) on AND detection showing faster “no” responses in the target-absent trials compared to “no” responses obtained from single-target trials. One may argue this is because observed “no” responses follow an OR rule in AND designs; that is, the correct response is “no” when a target is absent in position A, B, OR both A and B. Therefore, the presence of two non-targets affords more opportunities to detect absent information on target-absent “e” trials. Overall, this yields the prediction for faster “no” responses in absent (e) vs. single-target **A** or **B** only trials.

To assess preliminary predictions, simulations using the *sft.R package* were carried out (Houpt et al., 2014). First, a 3 × 3 design was simulated using the *sic.test* function by implementing a parallel independent-race model with an OR decision (min) rule to generate vectors of RTs in the D_1_ and D_2_ subgroup conditions. (Single-target conditions, M_1_/M_2_, were simulated using only one channel without an “absent” process being present). Empirical survivor functions of RTs were obtained from randomly generated exponential distributions. Default parameter settings for exponential rates governing high vs. low salience were used (channel A, λ_*HA*_ = 0.2, λ_*LA*_ = 0.1 and for channel B, λ_*HB*_ = 0.21, λ_*LB*_ = 0.11). The two-sided Kolmogorov-Smirnov (KS) test for the survivor interaction contrast required approximately 1,500 trials per double-target condition to detect significant positive departures from 0 at α= 0.05. (Similar results occurred for parallel models with an AND decision rule). An actual experiment using this design requires 18,000 total trials to obtain enough RTs in each condition.

Significantly, the suggested dominance tests from (i) (comparing processing in the D_2_ vs. single-target conditions) were tested using the *siDominance* function. Here, the two-sided KS tests on survivor functions required only 200 trials per condition for significance at α= 0.005. These results suggest that the simpler comparisons across conditions motivated by subgroup structures are potentially more powerful than traditional interaction contrast tests used for architecture identification.

#### 4.2.1 Extending tests to other decision rules

Example 4.4 provides just one example showing how breaking experimental conditions into subgroup components can inform data analyses to yield information regarding architecture.

In terms of other paradigms, one may consider testing predictions of XOR decision rules on architecture, or perhaps a rule allowing for separate Yes/No responses for each factor.

Finally, future research might derive predictions for other configurations that follow neither parallel nor serial networks. One exemplary architecture or directed graph that follows neither parallel nor serial logic gates is the Wheatstone bridge. In fact, any network with the properties of being (i) acyclic, (ii) existing on a directed graph, and (iii) with components operating neither in parallel nor serial is known to contain a Wheatstone bridge as a subcomponent (Dodin, 1985).

**Figure 2:**
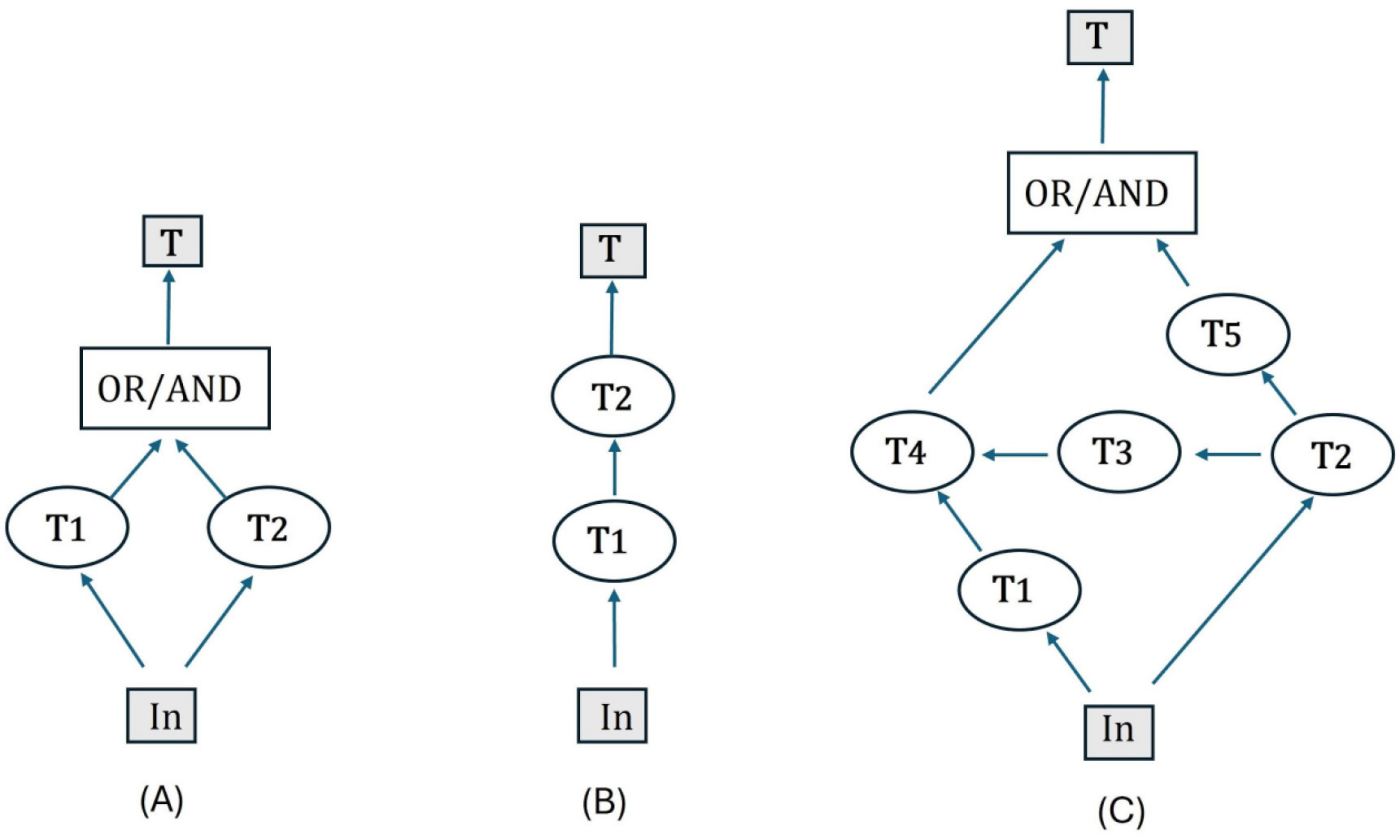
Assume a double-target input (In) is added to the model. **T**_**i**_ indicates processing times on each component. **(A)** A parallel model with OR or AND logic gate; **(B)** A serial model whose total processing time T is the sum of two components; **(C)** A Wheatstone bridge that is neither parallel nor serial. The random vector **T** represents the observable output vector.

For comparison purposes, Figure 2 shows parallel and serial models (3a and b) as directed graphs with an AND/OR logic gate, vs. a Wheatstone bridge (c) with five component processes. This latter situation requires an experiment with 5-factors likely at two salience levels. Notice first that the balanced Wheatstone bridge, minus component **T**_**3**_, comprises a model with **T**_**1**_ + **T**_**4**_ and **T**_**2**_ + **T**_**5**_ acting in parallel; hence, **T** = *min/max*{**T**_**2**_ + **T**_**5**_, **T**_**1**_ + **T**_**4**_}. However, when component **T**_**3**_ is present, the directed graph becomes more complex: **T** = *min/max*{**T**_**2**_+ **T**_**5**_, *min/max*{**T**_**1**_**, T**_**2**_+ **T**_**3**_} + **T**_**4**_}; consequently, the model containing all components is neither strictly parallel nor serial. Certain graph components, namely **T**_**2**_ and **T**_**4**_ are arranged sequentially. Interestingly, however, the composition rule indicates that they can behave as if arranged in parallel (Schweickert, 1978).

## 5 Conclusion

This study described the group theoretical structure inherent in full-factorial experimental designs. First, simple 3 × 3 designs were shown to be isomorphic to the p-elementary group ℤ_3_ × ℤ_3_. One observes that the subgroups were organized into disjoint target-absent and single-target vs. double-target conditions. These ideas were extended to include broader experimental designs and their isomorphism classes: I showed how 3 × 3 × 3 full-factorial designs are also isomorphic to the p-elementary group of order 27, namely ℤ_3_ × ℤ_3_ × ℤ_3_. While not an exhaustive list by any means, the applications showed how subgroup structures can inform experimental design and data analyses, particularly in fractional designs, and how to identify main effects and interactions using subgroup decomposition. Together, these efforts should generalize beyond the specific examples presented in this study and represent an approachable empirical extension of Bailey's (e.g., 1981; 1985) work.

## Figures and Tables

**Table:** 

Term	Definition
Abelian group	A mathematical group consists of a set, along with a binary operator, where closure and associativity hold. Further, the set must contain the identity element, and each element must have an inverse. ***Abelian*** groups are commutative groups.
Extraspecial group	A group G with prime (p) elements whose center, Z, is cyclic and of order p. The quotient G/Z is an elementary Abelian p-group. Intuitively, order matters; however, order effects typically reduce to something simple (i.e., by changing the sign).
Frattini subgroup	The intersection of all maximal subgroups of a group G.
Gaussian binomial coefficient	The polynomial over *q* with integer coefficients. It is commonly expressed as ${\left(\begin{array}{c} n\\ k \end{array}\right)}_{q}$. It counts the number *k* of subspaces of dimension *q* over a larger *n*-dimensional space.
Heavy subgroups	*n*-Heavy subgroups, in this context, refer to subgroups, refer to subgroups in which each group element, except for the identity, has *n* or more factors present (i.e., not set to “0” or absent).
Heisenberg group	A group of 3 × 3 upper triangular matrices (under matrix multiplication) commonly arising in quantum mechanical systems. Order of multiplication matters and often yields different outcomes.
Homomorphism	A function between groups (or algebraic structures such as vector spaces) that preserves key components of the structures.
Probability space	A triple, generically (Ω, **F**, **P**), consisting of i) Ω: the set of all possible outcomes of an experiment (e.g., the six sides of a die), ii) the sigma-algebra **F**, which informally is a collection of subsets of Ω that constitute “events” (e.g., three even numbers in a row), and a probability measure **P**, that assigns “wights” or probabilities to each event in **F**.

## Data Availability

The original contributions presented in the study are included in the article/supplementary material, further inquiries can be directed to the corresponding author.
