# Supplementary material for: The Abelian group structure of full factorial designs
Source: Front Psychol. 2026 May 20;17:1764159. doi: 10.3389/fpsyg.2026.1764159 (PMC13230083; doi:10.3389/fpsyg.2026.1764159)
Supplement: Supplementary file 1 [file Supplementary_file_1.docx]

**Appendix A**

**Lemma 2.1**: A full-factorial 3$\times$3 design, specified by the set of elements $S_{3\times3}$, is isomorphic to an Abelian group of order 9 under the operation of addition.

*Proof*: As in **Example 2.1**, $S_{3\times3}$contains nine elements, so all that is left to show is that it is describable as an Abelian group. One further observes that the elements of $S_{3\times3}$commute. Moreover, $A_{0}B_{0}$ will henceforth act as the identity element. Considering group criteria specified in the above paragraph, simple inspection shows that the addition of any two elements $A_{i}B_{j} \in S_{3\times3}$, and finally, each element has a unique inverse (as pointed out, each element besides the identity is order 3).$◻$

**Lemma 2.2**: All groups of order nine are Abelian.

*Proof*: By Lagrange’s theorem, the order of each element must divide the order of the group. The term “Order” refers to the number of times an element must be added (or multiplied if the operation is multiplication) to itself to equal the identity. This leaves two possibilities: 1) the group is cyclic and hence each non-identity element has an order that divides 9. Such a group is therefore Abelian group and hence isomorphic to $\mathbb{Z}_{9}$. Otherwise, 2) each non-identity element is order 3.

Assume then, that each non-identity element has order 3. For notational simplicity, choose elements $a,b \in\mathbf{G}$, s.t. *b* is not an element of the cycle of *a*; recall *a* is an element of order 3, meaning $a\oplus a\oplus a = e \left( \mathrm{identity} \right)$. Therefore, $a\oplus b$ cannot equal the identity since element *b* is not the inverse of *a*, where the inverse is obviously $a\oplus a = a^{-1}$. (Identical logic applies to multiplicative groups). Now, the goal is to show that $a\oplus b = b\oplus a$ for all group elements (henceforth $ba = ab$; the operator shall be omitted for simplicity). If equality holds, we are done. Three other possible cases follow provided we assume the group is not abelian, from which a contradiction shall be derived:

1. Assume $ba = a^{2}b^{2}$. Obviously $ba \neq b^{2}a^{2}$ since each element is order 3 by assumption. Since both *a* and *b* are order three, $a^{2}b^{2}=a^{-1}b^{-1}$ , which is the inverse of $ba$ which equals $\left( ab \right)^{-1}$. This means that the inverse of an element must be equal to itself, which is impossible unless i) the element is the identity, or ii) the element is order 2, which is a contradiction.
2. Let $ba=a^{2}b$. Under conjugation we get: $bab^{-1}=a^{2}bb^{-1}=a^{2}$. Hence, conjugation has the effect of squaring *a*. By squaring we get:

$$b^{2}\left( bab^{-1} \right)b^{-2} = b^{2}a^{2}b^{-2} = a^{8} = a^{2}.$$

Using the associative property of groups, one may observe:

$$b\left( ba \right)\left( ab^{-1} \right)b^{-1}=b\left( a^{2}b \right)\left( ab^{-1} \right)b^{-1}=ba^{2}\left( ba \right)b^{-1}b^{-1}$$

$$=ba^{2}a^{2}\left( bb^{-1} \right)b^{-1}=ba^{4}b^{-1}=\left( a^{4} \right)^{2}bb^{-1}=a^{8}=a^{2}$$

This, of course, constitutes a contradiction since no element of **G** is order 1 except the identity.

1. Finally, assume: $ba = ab^{2}$. A nearly identical proof to 2, (this time making use of $b^{-1}$under conjugation) yields an equivalent contradiction.

Therefore, $ba = ab$ in all cases. In conclusion, all groups of order 9 are Abelian. $◻$

**Theorem 2.1**: The full-factorial identification design, whose elements are specified by the set$S_{3\times3}$ , is isomorphic to the p-elementary Abelian group $\mathbb{Z}_{3}\times\mathbb{Z}_{3}$.

*Proof*: By Lemmas 2.1 and 2.2, observe that the group specified by $S_{3\times3}$ is abelian and order 9. Either $S_{3\times3}\cong\mathbb{Z}_{9}$ or $S_{3\times3} \cong\mathbb{Z}_{3}\times\mathbb{Z}_{3}$. The group is not isomorphic to $\mathbb{Z}_{9}$ since each non-identity element is order 3. Logically, this leaves the direct product of cyclic Abelian groups $\mathbb{Z}_{3}\times\mathbb{Z}_{3}$as the only possibility for an isomorphic relation. Further, the nine elements of $\mathbb{Z}_{3}\times\mathbb{Z}_{3}$ under group addition include {(0,0), (0,1), (0,2), (1,0), (2,0), (1,1), (1,2), (2,1), (2,2)}: these elements correspond bijectively to the conditions of the detection design specified by $S_{3\times3}$. Since the group tables are identical, we have established an isomorphism. Finally, $\mathbb{Z}_{3}\times\mathbb{Z}_{3}$ is a p-elementary group since p in $\mathbb{Z}_{p}\times\mathbb{Z}_{p}$ is prime, thereby constituting a direct product of cyclic groups of order three whose order is *p*^2^ $=$ 3^2^ $=$ 9. $◻$

**Theorem 3.2**: Consider the full-factorial design A_i_$\times$B_j_ $\times$…$\times$C_k_ $\cong$ to $\mathbb{Z}_{m}\times\mathbb{Z}_{n}\times...\times\mathbb{Z}_{r}$ . By Lemma 2.1; it can be shown to be isomorphic to an Abelian group: namely a direct product $Z_{m}\times Z_{n}\times... {\times Z}_{r}.$ Assume factor levels m, n, r, are integers not relatively prime. Then, there exists a p-elementary subgroup $\mathbb{Z}_{p}\times\mathbb{Z}_{p}\times...\times\mathbb{Z}_{p}$, with an identical number of factors as $\mathbb{Z}_{m}\times\mathbb{Z}_{n}\times...\times\mathbb{Z}_{r}$.

*Proof*: Any set of integers not relatively prime to one another must contain a greatest common divisor (gcd), d. If d is prime, we are done. Assuming d is not prime, then by the fundamental theorem of algebra d = p_1_$\times$p_2_$\times$…$\times$p_k_, where each p_i_ is prime though not necessarily distinct. Hence, each p_i_ divides every integer that specifies the number of levels for each factor. Therefore, there exists a *p* | m, n,…,r. Therefore, we have $\mathbb{Z}_{p}\times\mathbb{Z}_{p}\times...\times\mathbb{Z}_{p}$, which is a p-elementary subgroup of **G** of direct products whose order must divide **G** such that each group element is order *p*.$◻$

**Proposition** **3.4**: The number of trial types in which all targets are present (in p-elementary groups) is (p – 1)^k^.

*Proof*: This is straightforward. For three factors at three levels for instance, $\mathbb{Z}_{3}\times\mathbb{Z}_{3}\times\mathbb{Z}_{3}$, we get (3-1)^3^ = 8 redundant-target trial types. As an extension of the 3$\times$3 case involving a double difference, an interaction contrast function using 8 trial-types involves a double difference of a double difference, etc. Generalization follows by simple induction. $◻$

**Appendix B**

*Representative non-zero vectors (x, y, z)*

$$\left\{ \begin{matrix} \left( 1,0,0 \right) & \left( 0,1,0 \right) \\ \left( 0,0,1 \right) & \left( 1,1,0 \right) \\ \left( 1,2,0 \right) & \left( 1,0,1 \right) \\ \left( 1,0,2 \right) & \left( 0,1,1 \right) \\ \left( 0,1,2 \right) & \left( 1,1,1 \right) \\ \left( 1,1,2 \right) & \left( 1,2,1 \right) \\ \left( 1,2,2 \right) & \end{matrix} \right\}$$

*Subgroups of order 3*

$$\left\{ \begin{aligned} \left( 0,0,0 \right) \\ \left( 0,0,1 \right) \\ \left( 0,0,2 \right) \end{aligned} \right\}, \left\{ \begin{aligned} \left( 0,0,0 \right) \\ \left( 0,1,0 \right) \\ \left( 0,2,0 \right) \end{aligned} \right\}, \left\{ \begin{aligned} \left( 0,0,0 \right) \\ \left( 1,0,0 \right) \\ \left( 2,0,0 \right) \end{aligned} \right\} 1-Heavy$$

$$\left\{ \begin{aligned} \left( 0,0,0 \right) \\ \left( 2,2,0 \right) \\ \left( 1,1,0 \right) \end{aligned} \right\}, \left\{ \begin{aligned} \left( 0,0,0 \right) \\ \left( 2,1,0 \right) \\ \left( 1,2,0 \right) \end{aligned} \right\}, \left\{ \begin{aligned} \left( 0,0,0 \right) \\ \left( 0,1,2 \right) \\ \left( 0,2,1 \right) \end{aligned} \right\}, \left\{ \begin{aligned} \left( 0,0,0 \right) \\ \left( 0,2,2 \right) \\ \left( 0,1,1 \right) \end{aligned} \right\} ,\left\{ \begin{aligned} \left( 0,0,0 \right) \\ \left( 2,0,2 \right) \\ \left( 1,0,1 \right) \end{aligned} \right\}, \left\{ \begin{aligned} \left( 0,0,0 \right) \\ \left( 1,0,2 \right) \\ \left( 2,0,1 \right) \end{aligned} \right\} 2-Heavy$$

$$\left\{ \begin{aligned} \left( 0,0,0 \right) \\ \left( 1,1,1 \right) \\ \left( 2,2,2 \right) \end{aligned} \right\}, \left\{ \begin{aligned} \left( 0,0,0 \right) \\ \left( 2,2,1 \right) \\ \left( 1,1,2 \right) \end{aligned} \right\}, \left\{ \begin{aligned} \left( 0,0,0 \right) \\ \left( 1,2,2 \right) \\ \left( 2,1,1 \right) \end{aligned} \right\}, \left\{ \begin{aligned} \left( 0,0,0 \right) \\ \left( 1,2,1 \right) \\ \left( 2,1,2 \right) \end{aligned} \right\} 3-Heavy$$

*Subgroups of order 9*

(1,0,0): x = 0 (0,1,0): y = 0 (0,0,1): z = 0

$\left\{ \begin{aligned} \left( 0,0,0 \right) \\ \left( 0,1,0 \right) \\ \left( 0,2,0 \right) \\ \left( 0,0,1 \right) \\ \left( 0,0,2 \right) \\ \left( 0,1,1 \right) \\ \left( 0,1,2 \right) \\ \left( 0,2,1 \right) \\ \left( 0,2,2 \right) \end{aligned} \right\}$ $\left\{ \begin{aligned} \left( 0,0,0 \right) \\ \left( 1,0,0 \right) \\ \left( 2,0,0 \right) \\ \left( 0,0,1 \right) \\ \left( 0,0,2 \right) \\ \left( 1,0,1 \right) \\ \left( 1,0,2 \right) \\ \left( 2,0,1 \right) \\ \left( 2,0,2 \right) \end{aligned} \right\}$ $\left\{ \begin{aligned} \left( 0,0,0 \right) \\ \left( 1,0,0 \right) \\ \left( 2,0,0 \right) \\ \left( 0,1,0 \right) \\ \left( 0,2,0 \right) \\ \left( 1,1,0 \right) \\ \left( 1,2,0 \right) \\ \left( 2,1,0 \right) \\ \left( 2,2,0 \right) \end{aligned} \right\}$

(1,1,0): y = -x (1,2,0): y = x (1,0,1): z = -x (1,0,2): z = x (0,1,1): z = -y

$\left\{ \begin{aligned} \left( 0,0,0 \right) \\ \left( 0,0,1 \right) \\ \left( 0,0,2 \right) \\ \left( 1,2,0 \right) \\ \left( 1,2,1 \right) \\ \left( 1,2,2 \right) \\ \left( 2,1,0 \right) \\ \left( 2,1,1 \right) \\ \left( 2,1,2 \right) \end{aligned} \right\}$ $\left\{ \begin{aligned} \left( 0,0,0 \right) \\ \left( 0,0,1 \right) \\ \left( 0,0,2 \right) \\ \left( 1,1,0 \right) \\ \left( 1,1,1 \right) \\ \left( 1,1,2 \right) \\ \left( 2,2,0 \right) \\ \left( 2,2,1 \right) \\ \left( 2,2,2 \right) \end{aligned} \right\}$ $\left\{ \begin{aligned} \left( 0,0,0 \right) \\ \left( 0,1,0 \right) \\ \left( 0,2,0 \right) \\ \left( 1,0,2 \right) \\ \left( 1,1,2 \right) \\ \left( 1,2,2 \right) \\ \left( 2,0,1 \right) \\ \left( 2,1,1 \right) \\ \left( 2,2,2 \right) \end{aligned} \right\}$ $\left\{ \begin{aligned} \left( 0,0,0 \right) \\ \left( 0,1,0 \right) \\ \left( 0,2,0 \right) \\ \left( 1,0,1 \right) \\ \left( 1,1,1 \right) \\ \left( 1,2,1 \right) \\ \left( 2,0,2 \right) \\ \left( 2,1,2 \right) \\ \left( 2,2,2 \right) \end{aligned} \right\}$ $\left\{ \begin{aligned} \left( 0,0,0 \right) \\ \left( 1,0,0 \right) \\ \left( 2,0,0 \right) \\ \left( 0,1,2 \right) \\ \left( 1,1,2 \right) \\ \left( 2,1,2 \right) \\ \left( 0,2,1 \right) \\ \left( 1,2,1 \right) \\ \left( 2,2,1 \right) \end{aligned} \right\}$

(0,1,2): y +2z = 0 (1,1,1): x+y+z = 0 (1,1,2): x+y+2z = 0 (1,2,1): x+2y+z = 0

$\left\{ \begin{aligned} \left( 0,0,0 \right) \\ \left( 1,0,0 \right) \\ \left( 2,0,0 \right) \\ \left( 0,1,1 \right) \\ \left( 0,2,2 \right) \\ \left( 1,1,1 \right) \\ \left( 2,1,1 \right) \\ \left( 1,2,2 \right) \\ \left( 2,2,2 \right) \end{aligned} \right\}$ $\left\{ \begin{aligned} \left( 0,0,0 \right) \\ \left( 0,1,2 \right) \\ \left( 0,2,1 \right) \\ \left( 1,0,2 \right) \\ \left( 1,1,1 \right) \\ \left( 1,2,0 \right) \\ \left( 2,0,1 \right) \\ \left( 2,1,0 \right) \\ \left( 2,2,2 \right) \end{aligned} \right\}$ $\left\{ \begin{aligned} \left( 0,0,0 \right) \\ \left( 0,1,1 \right) \\ \left( 0,2,2 \right) \\ \left( 1,1,2 \right) \\ \left( 1,2,0 \right) \\ \left( 1,0,1 \right) \\ \left( 2,0,2 \right) \\ \left( 2,1,0 \right) \\ \left( 2,2,1 \right) \end{aligned} \right\}$ $\left\{ \begin{aligned} \left( 0,0,0 \right) \\ \left( 0,2,2 \right) \\ \left( 0,1,1 \right) \\ \left( 1,0,2 \right) \\ \left( 1,1,0 \right) \\ \left( 1,2,1 \right) \\ \left( 2,0,1 \right) \\ \left( 2,1,2 \right) \\ \left( 2,2,0 \right) \end{aligned} \right\}$

(1,2,2): x+2y+2z = 0

$\left\{ \begin{aligned} \left( 0,0,0 \right) \\ \left( 0,2,1 \right) \\ \left( 0,1,2 \right) \\ \left( 1,0,1 \right) \\ \left( 1,1,0 \right) \\ \left( 1,2,2 \right) \\ \left( 2,0,2 \right) \\ \left( 2,1,1 \right) \\ \left( 2,2,0 \right) \end{aligned} \right\}$
